# Microsomal epoxide hydrolase gene polymorphism and susceptibility to colon cancer

**DOI:** 10.1038/sj.bjc.6690028

**Published:** 1999-09-24

**Authors:** D J Harrison, A L Hubbard, J MacMillan, A H Wyllie, C A D Smith

**Affiliations:** CRC Laboratories, Department of Pathology, University of Edinburgh, Teviot Place, Edinburgh, EH8 9AG, UK

**Keywords:** epoxide hydrolase, colon cancer, polymorphism, cancer risk

## Abstract

We examined polymorphisms in exons 3 and 4 of microsomal epoxide hydrolase in 101 patients with colon cancer and compared the results with 203 control samples. The frequency of the exon 3 T to C mutation was higher in cancer patients than in controls (odds ratio 3.8; 95% confidence intervals 1.8–8.0). This sequence alteration changes tyrosine residue 113 to histidine and is associated with lower enzyme activity when expressed in vitro. This suggests that putative slow epoxide hydrolase activity may be a risk factor for colon cancer. This appears to be true for both right- and left-sided tumours, but was more apparent for tumours arising distally (odds ratio 4.1; 95% confidence limits 1.9–9.2). By contrast, there was no difference in prevalence of exon 4 A to G transition mutation in cancer vs controls. This mutation changes histidine residue 139 to arginine and produces increased enzyme activity. There was no association between epoxide hydrolase genotype and abnormalities of *p53* or Ki- *Ras*. © 1999 Cancer Research Campaign


British Journal of Cancer (1999) 79(1), 168–171
© 1999 Cancer Research Campaign

### Microsomal epoxide hydrolase gene polymorphism and susceptibility to colon cancer

**DJ Harrison, AL Hubbard, J MacMillan, AH Wyllie and CAD Smith**

CRC Laboratories, Department of Pathology, University of Edinburgh, Teviot Place, Edinburgh EH8 9AG, UK

**Summary** We examined polymorphisms in exons 3 and 4 of microsomal epoxide hydrolase in 101 patients with colon cancer and compared the results with 203 control samples. The frequency of the exon 3 T to C mutation was higher in cancer patients than in controls (odds ratio 3.8; 95% confidence intervals 1.8–8.0). This sequence alteration changes tyrosine residue 113 to histidine and is associated with lower enzyme activity when expressed in vitro. This suggests that putative slow epoxide hydrolase activity may be a risk factor for colon cancer. This appears to be true for both right- and left-sided tumours, but was more apparent for tumours arising distally (odds ratio 4.1; 95% confidence limits 1.9–9.2). By contrast, there was no difference in prevalence of exon 4 A to G transition mutation in cancer vs controls. This mutation changes histidine residue 139 to arginine and produces increased enzyme activity. There was no association between epoxide hydrolase genotype and abnormalities of *p53* or Ki-*Ras*.

**Keywords:** epoxide hydrolase; colon cancer; polymorphism; cancer risk

The colon is subject to oxidative and free radical damage by both products of endogenous metabolism and bacterial fermentation within the gut lumen. These injurious stimuli may cause cell damage or cell death or even lead to mutations, resulting in tumour initiation and progression. Colon cancer is a common disease, with the highest incidence in developed countries. The aetiology is unknown, but in addition to diet and cigarette smoking (Giovannucci and Willett, 1994) there is a complex polygenic background that determines individual susceptibility to disease. There is currently much interest in the roles of oncogenes, tumour-suppressor genes and mismatch repair enzymes in colon cancer (Tomlinson et al, 1997). In addition, interindividual variation in the ability to dispose of reactive xenobiotics catalysed by glutathione S-transferases GST-M1 and GST-T1 has been investigated (Lang et al, 1986; Strange et al, 1991; Zhong et al, 1993; Chenevix-Trench et al, 1995). However, results from a number of studies show only weak and inconsistent associations with disease susceptibility. *N*-acetyltransferase 2 (NAT-2) polymorphism may be implicated in susceptibility to colon cancer (Lang et al, 1986; Wohllebet al, 1990; Illett et al, 1994; Probst-Hensch et al, 1995), but there is evidence that its relationship may be by linkage with other genes rather than causally (Hubbard et al, 1997).

We have used a polymerase chain reaction (PCR) strategy to investigate whether polymorphisms in the microsomal epoxide hydrolase gene (mEPHX) (Hassett et al, 1994) have any relationship to colon cancer. The enzyme is expressed in many tissues, including colon and liver. Polymorphisms of mEPHX may have functional significance. There is variation in exon 3, where a T to C alteration changes tyrosine residue 113 to histidine and is associated with lower enzyme activity when expressed in vitro. By contrast, A to G transition in exon 4 changes histidine residue 139 to arginine and produces increased enzyme activity. The effect of combining the alleles has not been established. The activity of mEPHX varies more than 50-fold in Caucasians (Omiecinski et al, 1993). This variation of activity is, thus, due to a combination of genetic polymorphism, transcriptional and post-transcriptional control of gene expression.

### MATERIALS AND METHODS

### Controls and cancer cases

Control blood samples ($n = 203$) were obtained anonymously from the Scottish National Blood Transfusion Service. These were Caucasian individuals aged between 18 and 65 years with equal sex distribution. This group has been previously described (Cantlay et al, 1994) and was drawn from the same geographical area as the cancer study group. The presence of colorectal neoplasia was not specifically excluded, but all patients were healthy. Peripheral blood from patients with colorectal cancer was collected from a consecutive series of operable colorectal cancer cases after surgery in four local hospitals between 1988 and 1993 ($n = 101$).

### Cancer patient data

Cancer diagnosis was confirmed histopathologically and cases were classified according to Dukes' stages (A, B, C), and according to position of cancer in the colon as either right (caecum, transverse or ascending) or left (sigmoid, descending or rectum) sides. All samples were Caucasian in origin. DNA was extracted as previously described (Cantlay et al, 1995). In addition, the frequency of immunodetectable stabilization of *p53* was recorded using antibody DO7 (Dako) on formalin-fixed, paraffin-embedded tissue ($n = 92$). Loss of heterozygosity at the *p53* locus on chromosome 17 ($n = 93$) was determined as previously reported (Cripps, 1994). The presence of codon 12 mutations in Ki-*ras* oncogene was determined using allele-specific PCR ($n = 81$) as described previously (Kotsinas et al, 1993).

*Received 17 November 1997*
*Revised 11 May 1998*
*Accepted 13 May 1998*

*Correspondence to:* DJ Harrison

**Table 1** Distribution of microsomal epoxide hydrolase genotypes in control and disease populations

| | Exon 3 polymorphism | | | | | | Exon 4 polymorphism | | | | |
|---|---|---|---|---|---|---|---|---|---|---|---|
| | Tyr (%) | Tyr/His (%) | His (%) | $\chi^2$ (*P*-value) | OR (95% CI) | MAF | His (%) | His/Arg (%) | Arg (%) | $\chi^2$ (*P*-value) | MAF |
| Controls (*n* = 203) | 91 (45) | 99 (49) | 13 (6) | – | – | 0.31 | 147 (72) | 53 (26) | 3 (2) | – | 0.15 |
| Colorectal cancer (*n* = 101) | 42 (50) | 38 (40) | 21 (10) | 14.5 0.007 | 3.84 (1.83–8.04) | 0.40 | 76 (75) | 21 (21) | 4 (4) | 2.66 >0.10 | 0.15 |
| Right-sided (*n* = 33) | 11 (33) | 16 (49) | 6 (18) | 5.7 >0.10 | 3.25 (1.14–9.27) | 0.43 | 21 (64) | 9 (27) | 3 (9) | 6.79 >0.10 | 0.23 |
| Left-sided (*n* = 68) | 31 (46) | 22 (32) | 15 (22) | 15.2 0.002 | 4.10 (1.87–9.15) | 0.38 | 55 (81) | 12 (18) | 1 (1) | 2.0 >0.10 | 0.10 |

For exon 3 genotype, the designation 'Tyr' represents an individual carrying homozygous Tyr113 mEPHX sequence, 'His' represents a homozygous His-113 and 'Tyr/His' a heterozygote. For exon 4 genotype, the designation 'His' represents an individual carrying homozygous His-139 mEPHX sequence, 'Arg' represents a homozygous Arg-139 and 'His/Arg' a heterozygote. *P*-values for $\chi^2$ for right- and left-sided compared with controls are given as Bonferroni corrected. Odds ratios (OR) and 95% confidence intervals (CI), relative risk of the presence of two His-113 exon 3 alleles in disease population vs controls. MAF = 'mutant' allele frequency.

**Table 2** Distribution of mEPHX exon 3 genotypes and *p53* immunodetection, *p53* loss of heterozygosity and Ki-*ras* codon 12 mutation

| | Exon 3 polymorphism | | | |
|---|---|---|---|---|
| | Tyr (%) | Tyr/His (%) | His (%) | *P*-value |
| *p53* immunodetection (*n* = 92) | | | | |
| Positive | 17 (18) | 13 (14) | 9 (10) | >0.10 |
| Negative | 21 (23) | 22 (24) | 10 (11) | |
| *p53* loss of heterozygosity (*n* = 93) | | | | |
| Loss | 8 (9) | 9 (10) | 3 (3) | >0.10 |
| Retained | 11 (12) | 8 (9) | 4 (4) | |
| Non-informative | 21 (22) | 17 (18) | 12 (13) | |
| Ki-*Ras* mutation at codon 12 (*n* = 81) | | | | |
| Wild type | 7 (9) | 6 (7) | 5 (6) | >0.10 |
| Mutated | 28 (34) | 24 (30) | 11 (14) | |

Nomenclature of mEPHX exon 3 alleles as described in Table 1. *P*-value calculated from $\chi^2$ comparing exon 3 allele frequency within each group, for example p53 positive vs p53 negative.

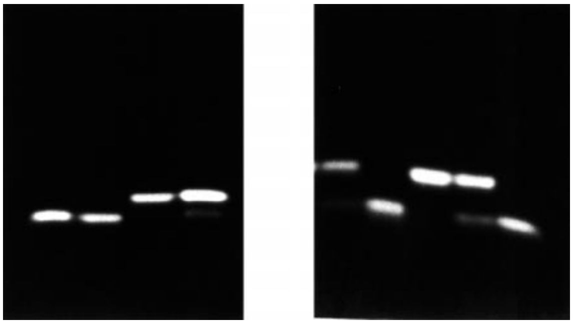

**Figure 1** Epoxide hydrolase

### mEPHX PCR analysis

Two separate PCR assays were used to detect the two mutations in mEPHX. The assay for the exon 3 T to C variant, changing tyrosine 113 to histidine, uses the primer pair E1 5′-GATCGATAAGTTC-CGTTTCACC [starting at bp 321 in mEPHX cDNA] and E2 5′-ATCCTTAGTCTTGAAGTGAGGaT (starting at bp 461). The downstream primer abuts directly onto the mutation site and an engineered base change (shown in lower case: an A for a G) produces an *Eco*RV restriction enzyme site (GATATC) in the wild type only. The exon 4 A to G transition produces an *Rsa*I restriction fragment length polymorphism (ATAC to GTAC). The primer pair E3 5′-ACATCCACTTCATCCACGT (bp 494) and E4 5′-ATGC-CTCTGAGAAGCCAT (bp 685) is used to assay this polymorphism. Figure 1 shows a typical result of the genotyping assays. Polymorphisms were detected using restriction enzymes *Eco*RV (exon 3) and *Rsa*I (exon 4). The polymerase chain reaction was performed on a Hybaid Omnigene thermal cycler using 200 ng of genomic DNA, 200 ng of primers E1/E2 or E3/E4, 200 mM dNTPs (Pharmacia, UK), ×1 polymerase buffer (Promega, UK), 1.5 mM magnesium chloride, 4% DMSO and 2 u of *Taq* Polymerase (Promega, UK) in a total volume of 50 μl. Twenty microlitres of each PCR reaction was digested with 5 u of the appropriate restriction enzyme (Gibco BRL, UK). Digested PCR products were separated by size on 1.8% Metaphor (Hoeffer Scientific) agarose gel. Bands were visualized by ethidium bromide staining and ultraviolet illumination. Main cycling parameters were: 38 cycles of 94°C for 30 s, 55°C for 25 s and 72°C for 20 s.

### Statistical analysis

Associations between disease groups and specific genotypes and phenotypes were analysed for significance by the two-tailed chi-squared test and *P*-values were Bonferroni corrected. Odds ratios and 95% confidence intervals were calculated to assess relative risk of disease conferred by a particular allele or genotype.

### RESULTS

### mEPHX gene analysis

Analysis of the control group showed that the exon 3 histidine 113 ('slow') form was threefold more common than the exon 4 arginine 139 ('fast') form in the Caucasian population; 13 (6%) out of

© Cancer Research Campaign 1999

203 individuals were homozygous for histidine 113 (exon 3) and 49% heterozygous. In comparison, arginine 139 (exon 4) was only detected in 28% of individuals, with only three homozygous subjects in total (Table 1). These results are similar to previous studies (Smith and Harrison, 1997).

### mEPHX polymorphisms in disease groups

The distribution of mEPHX genotypes are shown in Table 1, and the comparison of exon 3 alleles with *p53* and Ki-*ras* mutations shown in Table 2. In the group of colon cancer patients (Table 1), there was a significant increase in the proportion of individuals homozygous for the histidine 113 (exon 3) [$P = 0.007$; odds ratio (OR) = 3.84]. Twenty-one out of 101 patients (21%) were homozygous for the histidine 113 (exon 3, putative 'slow' activity) versus only 6% of controls. There was no significant difference in the distribution of arginine 139 (exon 4, putative 'fast' activity) between control and cancer groups. Cancers were divided into right- and left-sided groups and the association with histidine 113 polymorphism was recalculated. For right-sided tumours, there was a trend for the putative 'slow' allele to be more common in the cancer group, but this was not statistically significant when the Bonferroni correction was applied. By contrast, left-sided colon cancers showed a highly significant increase in histidine 113 'slow' allele compared with controls ($P = 0.002$; OR = 4.1). No association with sex, age or Dukes' stage was found for either allele (data not shown). No association between arginine 139 polymorphism and right- or left-sided tumours was identified. When compared with the frequency of immunodetectable stabilization of *p53*, loss of heterozygosity at the *p53* locus, or codon 12 mutation of Ki-*Ras*, no association was noted with polymorphisms of mEPHX at either exon 3 or exon 4 (Table 2). Comparisons of the observed distributions of mEPHX genotypes and those predicted by allele frequencies by chi-squared analysis showed that the populations studied were in Hardy–Weinberg equilibrium, indicating that the control and study groups were sufficiently random and representative (data not shown).

### DISCUSSION

The demonstration that genetically defined polymorphisms in mEPHX, predicted to affect enzyme activity at least in part, are associated with an increased incidence of colorectal cancer suggests that reactive epoxide intermediate metabolites may play a role in the development of colon cancer. Individuals with histidine 113 instead of tyrosine 113 (exon 3) had more than a threefold relative risk of having colorectal cancer. This is particularly true for cancers arising in the left side of the colorectum, i.e. descending and sigmoid colon and rectum, in which the relative risk increased to more than 4. The absence of a correlation with *p53* or Ki-*ras* mutations is unsurprising. mEPHX is a protective enzyme involved in general oxidative defence, rather than in specific protection of individual genes. Previously studies describing weak association between glutathione-dependent enzymes and colorectal cancer have found that the risk is more consistently with tumours originating in the left side (Zhong et al, 1993). This site may be more at risk from oxidative stress, or may be more likely to have high exposure to oxidants because of the higher transit time for faecal material at this site. Epoxides may be present in diet, or generated from a number of sources, including benzpyrene (which is present in cigarette smoke), dietary polycyclic aromatic hydrocarbons and nitrosamines (Craft et al, 1988; Yang et al, 1988). Some further evidence suggests that mEPHX may be involved in steroidogenesis reactions which may explain preliminary observations that 'slow' genotype may be protective for ovarian cancer (Lancaster et al, 1996) and is not involved in lung (Smith et al, 1997) or bladder (Brockmoller et al, 1996) cancer risk.

The present study has examined genotype and not phenotype. There is clear evidence that the presence of a 'slow' exon 3 allele does confer lower enzyme activity, but genotype alone is insufficient to explain the variation of microsomal epoxide hydrolase enzyme activity seen in population studies (Hassett et al, 1997). In particular, the effect of carrying both exon 3 and exon 4 polymorphisms is undetermined and, thus, assumptions concerning enzyme activity from our study should be necessarily guarded. It is still possible that the described mutations are of themselves not causally related to colon cancer, but rather are in linkage with other, as yet unidentified, factors. However, the clear association does indicate that this enzyme is an important candidate to relate diet with susceptibility of the colorectal mucosa to injury. Evidence that dietary supplements, particularly fish oils which are thought to be chemopreventative for colon cancer, can induce microsomal epoxide hydrolase (Yang et al, 1993) and, thus, increase enzyme activity further strengthens this association and indicates the need for further investigation.

### REFERENCES

Brockmoller J, Cascorbi I, Kerb R and Roots I (1996) Combined analysis of inherited polymorphisms in arylamine *N*-acetyltransferase 2, glutathione S-transferases M1 and T1, microsomal epoxide hydrolase, and cytochrome p450 enzymes as modulators of bladder cancer risk. *Cancer Res* **56**: 3915–3925

Cantlay AM, Smith CAD, Wallace WA, Yap P-L, Lamb D and Harrison DJ (1994) Heterogeneous and polymorphic expression of glutathione S-transferases in human lung. *Thorax* **49**: 1010–1014

Cantlay AM, Lamb D, Gillooly M, Norrman J, Morrison D, Smith CAD and Harrison DJ (1995) Association between the CYP1A1 gene polymorphism and susceptibility to emphysema and lung cancer. *J Clin Mol Pathol* **48**: M210–214

Chenevix-Trench G, Young J, Coggan M and Board O (1995) Glutathione S-transferase M1 and T1 polymorphisms: susceptibility to colon cancer and age of onset. *Carcinogenesis* **16**: 1655–1657

Craft JA, Bulleid NJ, Jackson MR and Burchell B (1988) Induction of microsomal epoxide hydrolase by nitrosamines in rat liver. Effect on messenger ribonucleic acids. *Biochem Pharmacol* **37**: 297–302

Cripps KJ, Purdie CA, Carder PJ, White S, Komine K, Bird CC and Wyllie AH (1994) A study of the stabilisation of p53 protein versus point mutation in colorectal carcinoma. *Oncogene* **9**: 2739–2743

Giovannucci E and Willett WC (1994) Dietary factors and risk of colon cancer. *Ann Med* **26**: 443–452

Hassett C, Aicher L, Sidhu LA and Omiecinski CJ (1994) Human microsomal epoxide hydrolase: genetic polymorphism and functional expression in vitro of amino acid variants. *Hum Mol Genet* **3**: 421–428

Hassett C, Lin J, Carty CL, Laurenzana EM and Omiecinski CJ (1997) Human hepatic microsomal epoxide hydrolase: comparative analysis of polymorphic expression. *Arch Biochem Biophys* **337**: 275–283

Hubbard AL, Harrison DJ, Moyes C, Wyllie AH, Cunningham C, Mannion E and Smith CAD (1997) *N*-acetyltransferase 2 genotype in colorectal cancer and selective gene retention in cancers with chromosome 8p deletions. *Gut* **41**: 229–234

Illett KF, Ingram DM, Carpenter DS, Teitel CH, Lang NP, Kadlubar FF and Minchin RF (1994) Expression of monomorphic and polymorphic *N*-acetyltransferases in human colon. *Biochem Pharmacol* **47**: 914–917

Kotsinas A, Spandidos DA, Romanowski P and Wyllie AH (1993) Relative expression of wild-type and activated Ki-ras2 oncogene in colorectal carcinomas. *Int J Oncol* **3**: 841–845

Lancaster JM, Brownlee HA, Bell DA, Futreal PA, Marks JR, Berchuck A, Wiseman RW and Taylor JA (1996) Microsomal epoxide hydrolase polymorphism as a risk factor for ovarian cancer. *Mol Carcinog* **17**: 160–162

© Cancer Research Campaign 1999

Lang NP, Chu DZJ, Hunter CF, Kendall DC, Flammang TJ and Kadlubar FF (1986) Role of aromatic amine *N*-acetyltransferase in human colorectal carcinoma. *Arch Surg* **121**: 1259–1261

Omiecinski CJ, Aicher L, Holubkov R and Checkoway H (1993) Human peripheral lymphocytes as indicators of microsomal epoxide hydrolase activity in liver and lung. *Pharmacogenetics* **3**: 150–158

Probst-Hensch NM, Haile RW, Ingles SA, Longnecker MP, Han C, Lin BK, Lee DB, Sakamoto GT, Frankl HD, Lee ER and Lin HJ (1995) Acetylation polymorphism and prevalence of colorectal adenomas. *Cancer Res* **55**: 2017–2020

Smith CAD and Harrison DJ (1997) Association between polymorphism in gene for microsomal epoxide hydrolase and susceptibility to emphysema. *Lancet* **350**: 630–633

Strange RC, Matharoo B, Faulder GC, Jones P, Cotton W, Elder JB and Deakin M (1991) The human glutathione S-transferases: a case control study of the incidence of the GST1 0 phenotype in patients with adenocarcinoma. *Carcinogenesis* **12**: 23–28

Tomlinson I, Ilyas M and Novelli M (1997) Molecular genetics of colon cancer. *Cancer Metastasis Rev* **16**: 67–79

Wohlleb JC, Hunter CF, Blass B, Kadlubar FF, Chu DZJ and Lang NP (1990) Aromatic amine acetyltransferase as a marker for colorectal cancer: environmental and demographic associations. *Int J Cancer* **46**: 22–30

Yang SK (1988) Sterioselectivity of cytochrome p-450 isozymes and epoxide hydrolase in the metabolism of polycyclic aromatic hydrocarbons. *Biochem Pharmacol* **37**: 61–70

Yang EK, Radominska A, Winder BS and Dannenberg AJ (1993) Dietary lipids coinduce xenobiotic metabolising enzymes in rat liver. *Biochim Biophys Acta* **1168**: 52–58

Zhong S, Wyllie AH, Barnes D, Wolf CR and Spurr NK (1993) Relationship between the GSTM1 genetic polymorphism and susceptibility to bladder, breast and colon cancer. *Carcinogenesis* **14**: 1821–1824

*© Cancer Research Campaign 1999*
